# West Nile Virus Meningitis in Patient with Common Variable Immunodeficiency

**DOI:** 10.3201/eid0910.030195

**Published:** 2003-10

**Authors:** Augusto M. Alonto, David M. Aronoff, Preeti N. Malani

**Affiliations:** * University of Michigan Health System, Ann Arbor, Michigan, USA

**To the Editor:** Infection by West Nile virus (WNV) was first recognized in the Western Hemisphere in 1999 in New York (*1*). Subsequently, this mosquito-borne flavivirus has spread westward and has emerged as an important cause of infectious meningoencephalitis in the United States (*2*). In September 2002, during a WNV epidemic in Michigan (*2*), a 38-year-old woman with common variable immunodeficiency (CVID) sought treatment at the University of Michigan Hospital with acute WNV-associated meningitis. Although persons with CVID are at increased risk for enteroviral meningoencephalitis, a greater susceptibility to arthropod-borne flavivirus infections has not been reported.

The patient had a history of recurrent sino-pulmonary infections and gastrointestinal giardiasis and salmonellosis; at 33 years of age, she was diagnosed with CVID that has been subsequently treated with intravenous immunoglobulin (IVIG) every 3 weeks. She was in her usual state of health until 5 days before admission, when she noted the abrupt onset of severe headache, followed by temperatures up to 39.4°C, progressive photophobia, nausea, vomiting, and a transient papular rash on her trunk and extremities.

On arrival, the patient reported marked photophobia. Physical examination showed a temperature of 40.6°C, heart rate 80 beats per minute, and blood pressure 122/70 mmHg. She had cervical tenderness to palpation and active range of motion with minimal rigidity. Small, diffuse, nontender lymphadenopathy was noted in the cervical region. No focal or global deficits were found on neurologic exam. Results of the remainder of the physical examination was unremarkable. Initial laboratory values included a peripheral leukocyte count of 3.5 K/mm^3^ (normal: 4.0–10.0 K/ mm^3^; 42% neutrophils, 52% lymphocytes, and 7% monocytes), which was unchanged from the patient’s baseline leukopenia. Her serum IgG level was 1,081 mg/dL (620–1,520 mg/dL).

Cerebrospinal fluid (CSF) sampling indicated the following: erythrocytes 3/mm^3^, leukocytes 77/mm^3^ (41% neutrophils, 51% lymphocytes, 7% histiocytes), glucose 50 mg/dL (50–70 mg/dL), and protein 75 mg/dL (15–45 mg/dL). Results of routine Gram stain, bacterial and fungal cultures, polymerase chain reaction testing for herpes simplex virus and *Cryptococcus neoformans* antigen were negative. Assay results of the patient’s CSF for WNV by IgM-capture enzyme-linked immunosorbent assay, performed by the Michigan Department of Community Health, were positive.

The patient was initially treated with parenteral ampicillin, ceftazidime, and acyclovir, which were discontinued within 48 hours. Her symptoms improved with routine medical support, and she was discharged on hospital day 5. We were notified of the positive CSF IgM for WNV approximately 2 weeks after the patient was discharged, at which time her symptoms had completely resolved. At a follow-up visit 3 weeks after her hospitalization, the patient had no residual symptoms of meningitis.

Patients with agammaglobulinemia, either common variable or X-linked, are known to be susceptible to recurrent infections (*3*). Bacterial infections are the best described; however, chronic enteroviral meningoencephalitis is also associated with deficiencies in B-cell immunity (*4*–*6*). Although the role of immunoglobulins in host defense against WNV infection is not completely understood, evidence suggests that humoral immunity protects against WNV infection and severe disease (*7*–*9*).

WNV is a single-stranded RNA virus of the family *Flaviviridae*. Its genome is processed to eight proteins, including the envelope (E) glycoprotein, the matrix protein, the nucleocapsid protein, and five nonstructural proteins (*7*). E-glycoprotein antibodies develop during human WNV infection (*8*), and passive immunization of mice with E-glycoprotein antiserum protects against WNV infection and death (*7*,*8*). In addition, IVIG therapy was associated with recovery from WNV meningoencephalitis of an immunosuppressed 70-year-old woman (*9*). Although our patient had normal levels of serum IgG at the time of illness, viral meningitis may occur in agammaglobulinemic patients despite regular IVIG therapy (*10*).

This case demonstrates the need to consider WNV in patients with CVID. Our patient recovered promptly, without evidence of neurologic sequelae, despite her underlying immunodeficiency. More experience is needed to provide a better understanding of the relationship between CVID and WNV.
